# Elevated Soluble ST2 Blood Levels in Patients with Depression and Comorbid Heart Failure: A Correlative Study

**DOI:** 10.1192/j.eurpsy.2024.1028

**Published:** 2024-08-27

**Authors:** A. K. Sikora, S. Fedorov

**Affiliations:** Ivano-Frankivsk National Medical University, Ivano-Frankivsk, Ukraine

## Abstract

**Introduction:**

Depressive disorders frequently coexist with chronic medical conditions like heart failure (HF), significantly impacting patients’ overall health and quality of life. This study aims to explore the correlation between soluble ST2 molecule levels and the presence of depressive disorders in patients with heart failure.

**Objectives:**

A total of 200 patients, all diagnosed with heart failure, were included in this study. Among them, 30 patients were mentally healthy, and the remaining 170 exhibited medium-level depressive disorders. Blood samples were collected and analyzed for soluble ST2 levels to assess the potential correlation between depressive disorders and soluble ST2 levels in patients with heart failure.

**Methods:**

A total of 200 patients, all diagnosed with heart failure, were included in this study. Among them, 30 patients were mentally healthy, and the remaining 170 exhibited medium-level depressive disorders. Blood samples were collected and analyzed for soluble ST2 levels to assess the potential correlation between depressive disorders and soluble ST2 levels in patients with heart failure.

**Results:**

The study demonstrated a statistically significant finding, indicating that the levels of soluble ST2 were 1.6 times higher in patients with depression and comorbid heart failure compared to mentally healthy individuals with heart failure.

**Conclusions:**

This study elucidates a statistically significant correlation between medium-level depressive disorders and elevated soluble ST2 levels in patients with coexisting heart failure, shedding light on the potential role of soluble ST2 as a biomarker in identifying and managing depressive disorders in heart failure patients. The observed 1.6-fold increase in soluble ST2 levels in heart failure patients with depression emphasizes the importance of mental health assessment and intervention in individuals with chronic medical conditions, particularly heart failure, to enhance overall care and outcomes.

**Disclosure of Interest:**

None Declared

